# Renalase Levels are Decreased in Maternal Blood and Placental Tissues in Pregnancies Associated with Preterm Preeclampsia

**DOI:** 10.21203/rs.3.rs-4319658/v1

**Published:** 2024-05-06

**Authors:** Youstina Soliman, Chino Eke, Xiaojia Guo, Melinda Wang, Tatiana Silva, Gary V. Désir, Liza Konnikova

**Affiliations:** 1 Department of Pediatrics, New Haven, CT, USA.; 2 Obstetrics, Gynecology and Reproductive Sciences, New Haven, CT, USA.; 3 Internal Medicine, New Haven, CT, USA.; 4 Immunobiology Yale University School of Medicine, New Haven, CT, USA.; 5 Department of Medical Sciences Frank H. Netter MD School of Medicine at Quinnipiac University, North Haven, CT, USA.; 6 Department of Internal Medicine, University of California San Francisco, San Francisco, CA.; 7 VA CT Medical Center, Yale University, New Haven, CT, USA.

**Keywords:** PEC, Preeclampsia, RNLS, Renalase, Placenta, Maternal Serum

## Abstract

Preeclampsia (PEC) is a complication of pregnancy associated with hypertension and the risk of eclampsia. The pathophysiology of PEC is unknown and identifying factors associated with PEC during pregnancy is crucial for placental, fetal, and maternal health. Renalase (RNLS) is an anti-inflammatory secretory flavoprotein associated with hypertension. Recent data demonstrated a correlation between maternal serum RNLS and PEC, and work from our group identified RNLS expression in the placenta. However, it remains unknown whether RNLS levels in placenta are altered by preeclampsia. Additionally, it is unclear if there is a differential effect of preterm and term PEC on RNLS. We demonstrate that serum RNLS was reduced in preterm cases of PEC. Similarly, placental RNLS was diminished in the chorion of preterm cases of PEC. However, a reduction of RNLS in the decidua was observed with all cases of PEC, while the levels of RNLS within the placental villi were similar in all cases. Overall, we demonstrate that RNLS correlates with PEC both systemically in maternal serum and locally within the placenta, with variable effects on the different layers of the placenta and more pronounced in preterm cases.

## Introduction

Preeclampsia (PEC) is a severe complication of pregnancy that affects 3 to 5% of global pregnancies and is one of the four leading causes of maternal death in the United States^1,2^. Although the exact mechanism remains elusive, the disease emanates from the maternal-fetal interface and is characterized by hypertension alongside proteinuria after 20-week gestation, and in severe cases, progresses to eclampsia^2,3^. For the fetus, preeclampsia can lead to growth restriction, oligohydramnios, and preterm birth^4,5^. The only known definitive treatment for PEC is delivery^1^. However, anti-hypertensive treatments that regulate blood pressure can ameliorate the symptoms^3^.

Renalase (RNLS) is an anti-inflammatory secretory flavoprotein present in the plasma initially identified in the kidneys. It has since been found in several tissues, including the heart^6^, pancreas^7^, and most recently by our group in the placenta^8^. Previous studies have demonstrated that RNLS levels are reduced in hypertension and kidney disease. Interestingly, several studies have suggested a reduction in maternal serum levels of RNLS in preeclampsia^9–12^. Our group demonstrated that RNLS is expressed in all layers of the placenta, with a notable abundance in trophoblasts and Hoffbauer cells, specialized placental macrophages^8^. However, it remains unknown whether RNLS level in placental tissue is altered in preeclampsia and if this is specific to the various layers of the placenta. Moreover, it is also unclear if RNLS is differentially expressed in the maternal serum and placental tissue of preterm and term preeclampsia cases.

The placenta is a critical transient organ that develops during early gestation to support the growing fetus. As the interface between the mother and fetus, it regulates the exchange of nutrients, hormones, and growth and immunologic factors necessary for maintaining a healthy pregnancy^13,14^. The placenta develops in a highly organized fashion, regulated by several hormones whose expression changes throughout pregnancy^14^. Dysregulation in placental development can lead to abnormal placentation during early gestation increasing the likelihood of perinatal morbidity and mortality^14,15^.

This temporary, but vital organ, is composed of several layers that ensure its proper function. The fetal membranes include the amnion and chorion derived from the chorionic sac while the maternal membrane is known as the decidua^16^. The chorion consists of a membrane bilayer composed of trophectoderm and the extraembryonic mesoderm which forms the barrier separating the maternal intervillous space from fetal circulation^13,17^. The decidua is comprised primarily of endometrial stromal cells and maternal vascular cells^18^. Finally, the placental villi consists of syncytiotrophoblasts, cytotrophoblasts, and villous core stromal cells^19^ and exists between the fetal membranes and the decidua forming a highly vascularized space that enables the bidirectional exchange of resources between the mother and fetus^20^.

In the current study, we explore if PEC correlates with RNLS levels in the maternal serum, the decidua, placental villi, or the fetal membranes, investigations that have not been previously carried out. To do so, we performed ELISAs on a cohort of 28 maternal serum samples and immunohistochemistry on 19 cases of preterm and term births with and without preeclampsia. By combining maternal samples with the fetus derived placenta, we hope to further elucidate the impact of PEC on RNLS. Our findings, consistent with previous studies, suggest that maternal serum RNLS levels are reduced in cases of preeclampsia. Interestingly, this was driven by the acid-sensitive fraction of serum RNLS and limited to preterm cases of PEC. Furthermore, while PEC diagnosis did not correlate with levels of RNLS within the placental villi, in the decidua and chorion layers RNLS levels were significantly reduced in preterm PEC compared to unaffected preterm deliveries. These findings help identify the potential correlation of maternal and fetal endogenous RNLS with the pathological development of the placenta and may suggest a possible therapeutic target for preeclampsia.

## Results

### RNLS level reduction in maternal serum is limited to preterm PEC.

**Figure 1.**
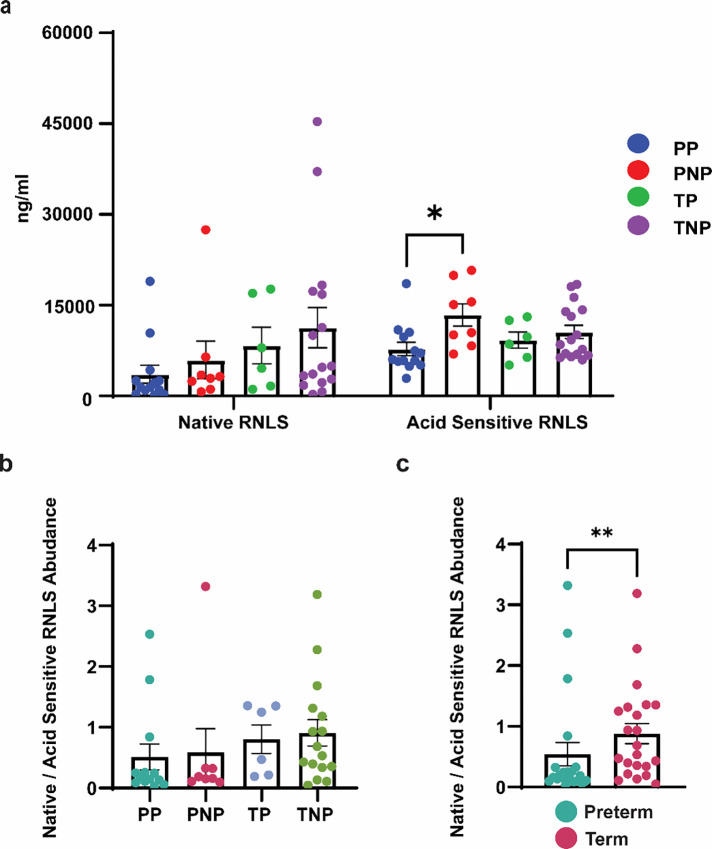
RNLS levels reduced in maternal serum in preterm cases with PEC. **(A)** ELISAs performed on 28 maternal serum samples display the RNLS abundance (ng/ml) for the native and acid sensitive fractions. **(B)** The ratio native RNLS to acid sensitive RNLS present in maternal serum per each cohort – PP, PNP, TP, and TNP. **(C)** Grouped ratio of native RNLS to acid sensitive RNLS of preterm and term samples. * Reflects a p-value < 0.05 and ** reflects a p-value < 0.01 PP – preterm preeclampsia, PNP – preterm no preeclampsia, TP – term preeclampsia, and TNP – term non preeclampsia.

To investigate the role of RNLS in PEC, we measured the concentration of RNLS (ng/ml) using our validated ELISA in maternal serum samples from 28 patients^21^. These patients either had healthy pregnancies or the diagnosis of PEC and experienced either a preterm or term delivery. Samples were grouped into four cohorts: preterm preeclampsia (PP, n = 7), preterm non-preeclampsia (PNP, n = 6), term preeclampsia (TP, n = 4), and term non-preeclampsia (TNP, n = 11) that were sex and gestational age balanced (**Table S1**). ELISA was performed on samples to determine the abundance of the two forms of RNLS, acid sensitive (serum bound fraction) and native (unbound fraction) RNLS, between the four groups. We observed a significant reduction in the acid sensitive RNLS concentration but not the native RNLS in preterm preeclampsia (PP) serum compared to preterm no preeclampsia (PNP) serum without a difference between term samples (Fig. 1A). The relative proportion of native and acid sensitive RNLS fractions in maternal serum was associated with the length of gestation. There was a higher concentration of acid sensitive RNLS in preterm deliveries compared to term deliveries while the proportion of native RNLS was higher in term than preterm cases, independent of a PEC diagnosis (Fig. 1B, C).

### RNLS levels vary between placental layers.

**Figure 2.**
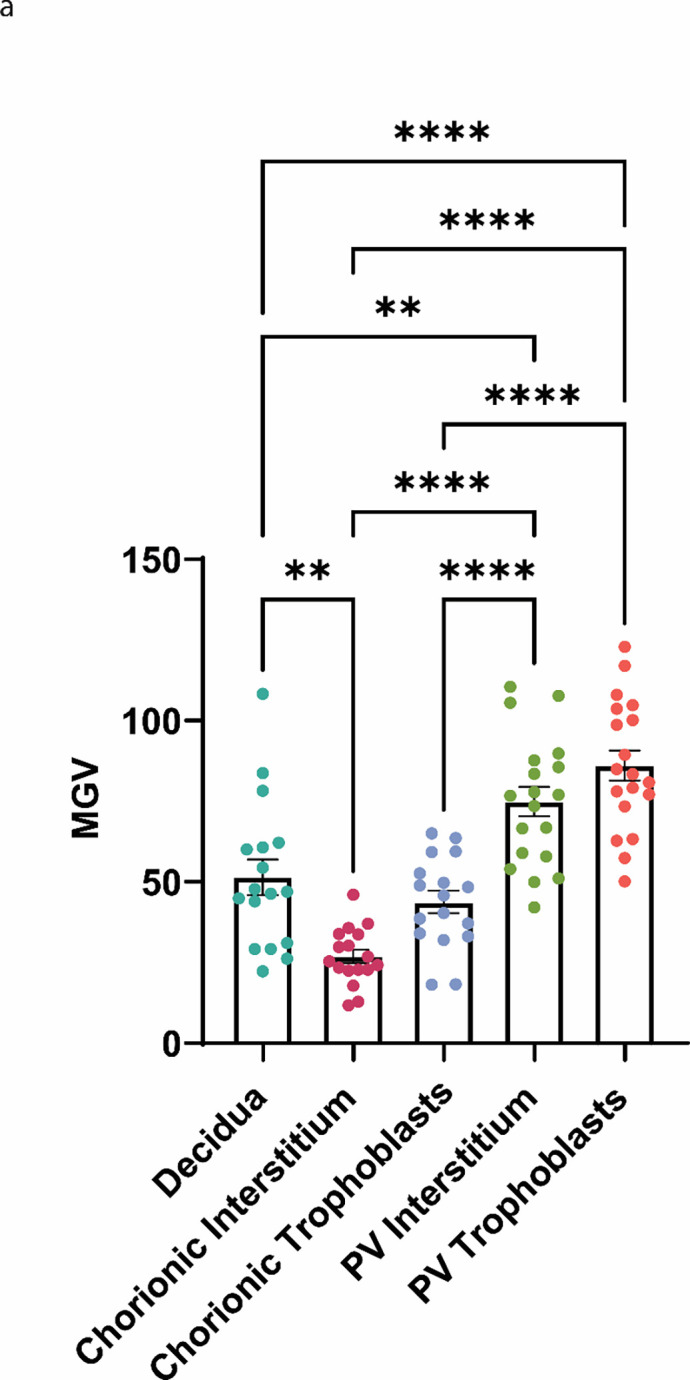
RNLS levels compared across placental layers and elevated in placental villi. **(A)** The RNLS expression per high powered field comparing decidua, chorionic interstitium, chorionic trophoblasts, placental villi interstitium, and placental villi trophoblasts. ** Reflects a p-value < 0.01 and **** reflects a p-value < 0.0001.

To further explore where RNLS is expressed in the placenta and if there is a correlation between PEC and RNLS levels in the placental tissue beyond maternal serum, we collected 19 placentas from preterm and term pregnancies with and without PEC. Samples were grouped into four cohorts: preterm preeclampsia (PP, n = 5), preterm non-preeclampsia (PNP, n = 4), term preeclampsia (TP, n = 5), and term non-preeclampsia (TNP, n = 5) which were well balanced based on sex and gestational age (**Table S2**). The placental tissue was separated into the decidua, chorion and placental villi components. RNLS levels were labeled via an immunohistochemistry (IHC) stain with our m28-RNLS antibody. When the mean grey values (MGVs) were compared across the various layers of the placenta, both the interstitium and trophoblast layers of the placental villi express significantly higher RNLS than both the decidua and chorionic components. Notably, no significant differences were observed between interstitium and trophoblast sections of either the chorion or the placental villi. (Fig. 2A). Considering the average MGV across the placental layers between the PP, PNP, TP, and TNP cohorts, similar trends were observed between layers within a particular cohort (**Table S3**).

### RNLS levels are reduced in the decidua affected by PEC.

**Figure 3.**
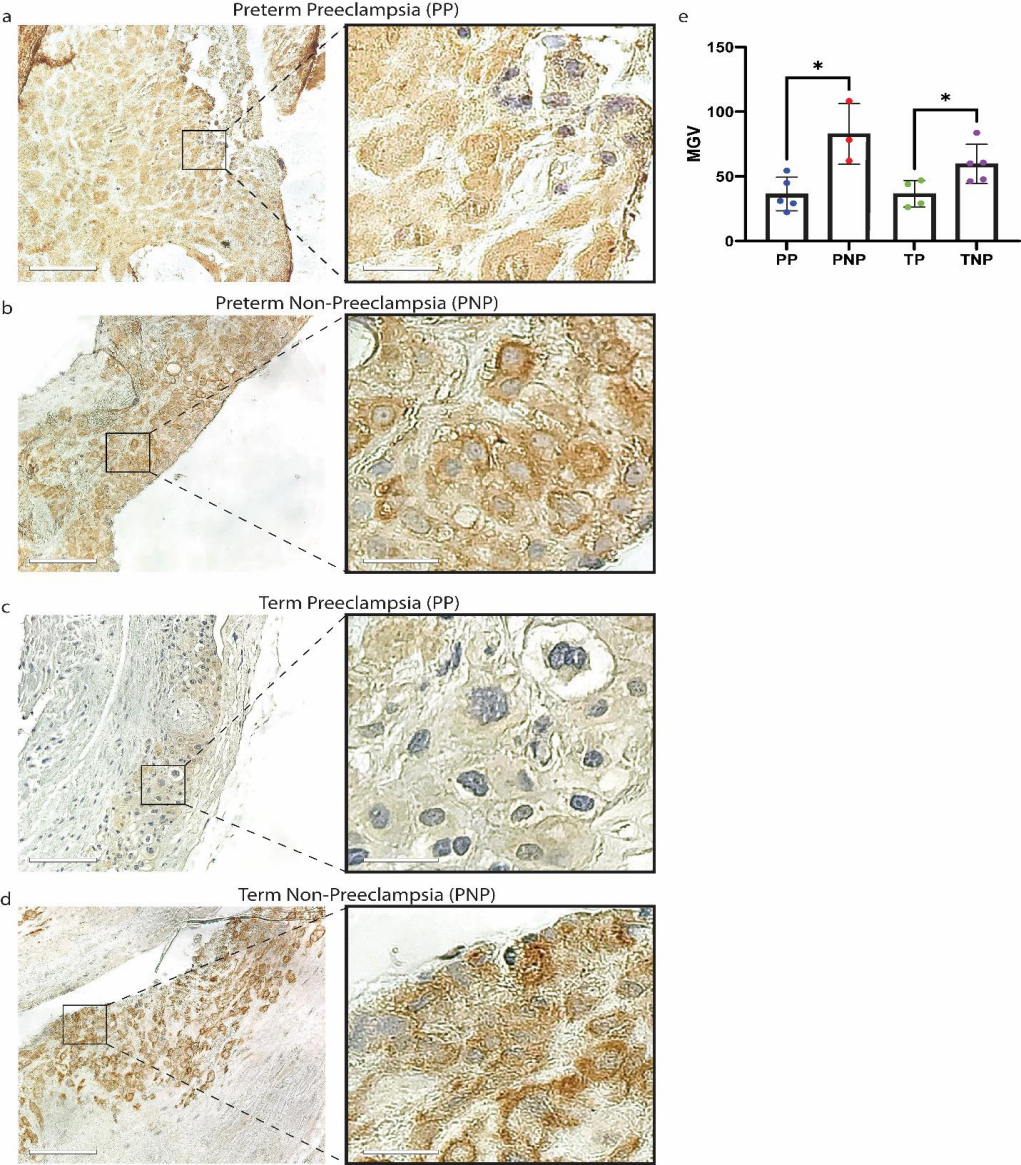
RNLS levels are reduced in the decidua samples affected by PEC. **(A-D)** Labeling for RNLS with m28 antibodies in decidual samples. Scale bar is 110 μm. Scale bar for inset is equivalent to 22 μm. **(E)** The RNLS expression per high powered field comparing PP, PNP, TP, and TNP in the decidua. * Reflects a p-value < 0.05. PP – preterm preeclampsia, PNP – preterm no preeclampsia, TP – term preeclampsia, and TNP – term non preeclampsia.

From the representative images of the decidua in the four groups, it appears that the labeling in the preeclampsia groups was lighter than in those resulting from pregnancies not effected by PEC (Fig. 3A–D). Upon quantification of the labeling intensities, we observed a significant reduction in RNLS levels amongst placentas of patients diagnosed with PEC (Fig. 3E). Interestingly, unlike in the maternal serum, where RNLS levels were only reduced in preterm PEC cases, with in the decidua, RNLS levels were reduced in both preterm and term deliveries effected by PEC (Fig. 3E).

### RNLS levels within the chorionic layer of the placenta are decreased in preterm cases of PEC.

**Figure 4.**
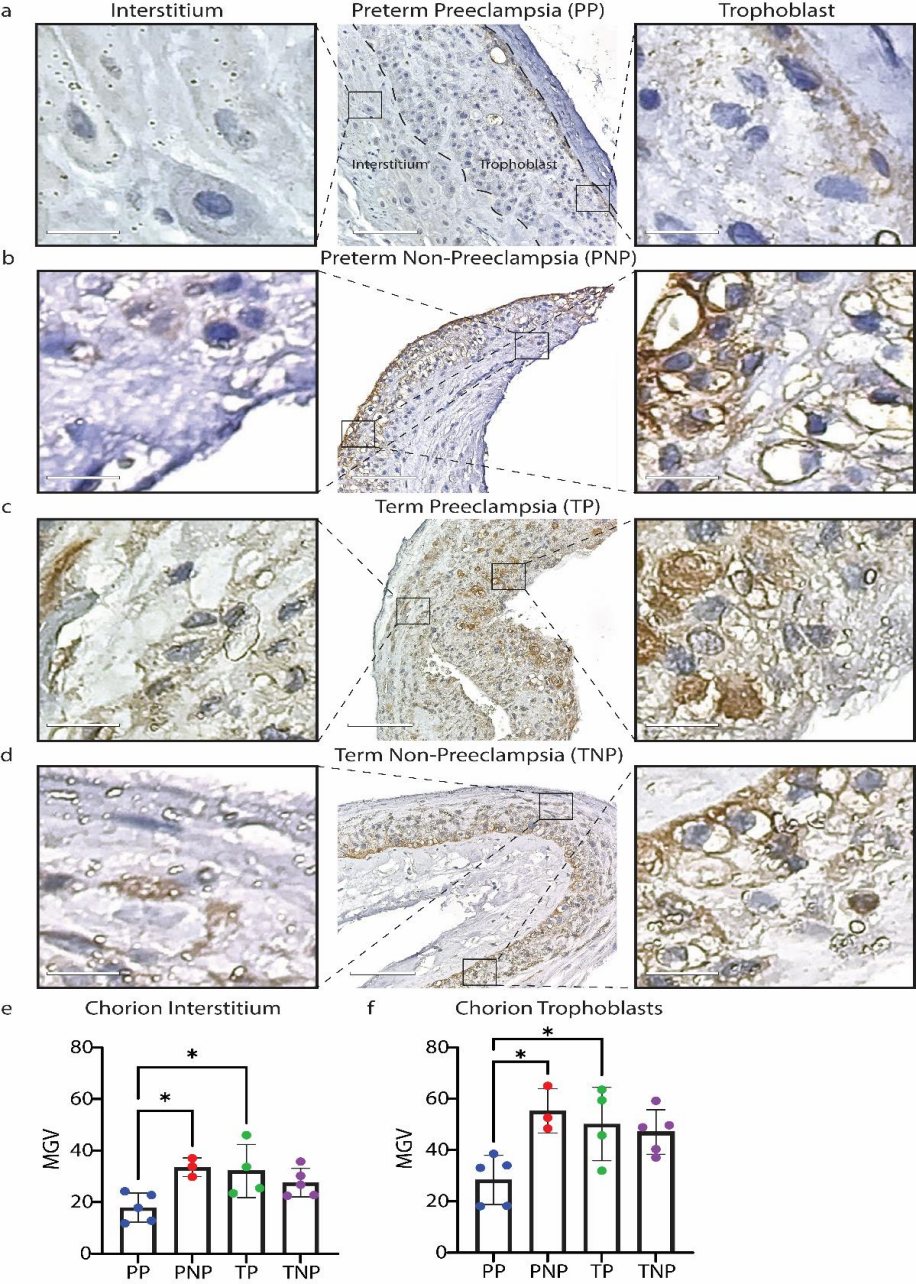
RNLS levels are reduced in the fetal membranes of preterm samples with PEC. **(A-D)** Labeling for RNLS with m28 antibodies in fetal membranes samples. Scale bar is 110 μm. Scale bar for inset is 15 μm. **(E)** The RNLS expression per high powered field comparing PP, PNP, TP, and TNP in the chorionic interstitium. **(F)** The RNLS expression per high powered field comparing PP, PNP, TP, and TNP in the chorionic trophoblast. * Reflects a p-value < 0.05. PP – preterm preeclampsia, PNP – preterm no preeclampsia, TP – term preeclampsia, and TNP – term non preeclampsia.

We then examined if RNLS levels are altered within the fetal membranes in preterm and full-term cases effected by PEC. Visually, there was a reduction in the levels of RNLS in the cases of preterm deliveries affected by PEC compared to all other cases (Fig. 4A–D). This was confirmed by the quantification of the RNLS labeling that demonstrated a significant reduction in RNLS levels in preterm cases effected by PEC but not the term cases (Fig. 4E). This was particularly driven by the levels of RNLS in the chorionic trophoblasts (Fig. 4F). Notably, the RNLS expression of the PP cohort is ~2x less than the RNLS expression of the PNP cohort in both the chorionic interstitium and chorionic trophoblasts (**Table S3**).

### PEC diagnosis is not associated with variable RNLS levels in the placental villi.

**Figure 5.**
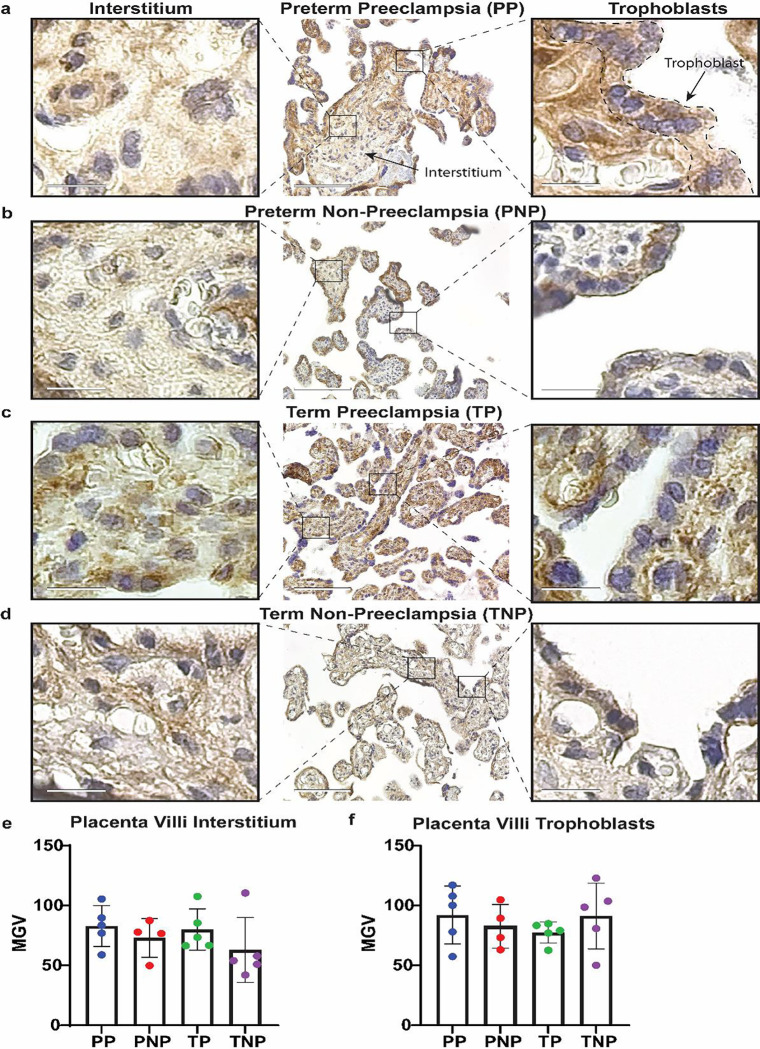
Placental Villi (PV) RNLS levels are not altered by PEC. **(A-D)** Labeling for RNLS with m28 antibodies in PV samples at 20x. Scale bar is equivalent to 110 μm. Scale bar for inset is equivalent to 15 μm. **(E)** The RNLS expression per high powered field comparing PP, PNP, TP, and TNP in the placenta villi interstitium. **(F)** The RNLS expression per high powered field comparing PP, PNP, TP, and TNP in the placenta villi trophoblast. * Reflects a p-value < 0.05. PP – preterm preeclampsia, PNP – preterm no preeclampsia, TP – term preeclampsia, and TNP – term non preeclampsia.

Finally, we investigated if the levels of RNLS were altered within the placental villi (PV). Similar to our previously published results, we were able to detect RNLS both in the PV trophoblasts and in the interstitium of the PV^8^. Visually, we did not observe any difference in the amount of RNLS present with in the PV between any of the four cohorts (Fig. 5A–D). This was confirmed upon quantification of the labeling, where there was no observed difference in RNLS levels between PP, PNP, TP, and TNP groups (Fig. 5E). This was also the case when we examined the trophoblast and interstitial components of the PV separately (Fig. 5E, F).

## Discussion

Preeclampsia (PEC) is a placental disease associated with hypertension, proteinuria, signs of uteroplacental dysfunction, or end-organ damage that can result in many complications for the maternal-fetal dyad alongside maternal risk of eclampsia^1,2,7^. Previous studies have shown an association between PEC and shallow placentation, systemic inflammation, and oxidative stress^7^. Given that the only known cure for PEC is the delivery of the fetus^1^, our investigation aimed to identify novel factors that influence the pathophysiology of PEC.

Renalase (RNLS) is a secretory flavoprotein that was initially identified to be secreted by the kidneys^7^ and has since been shown to be produced by several other organs^6–8^. It functions as a pro-survival, anti-inflammatory, growth-promoting factor with cellular antioxidative properties^10^. It also has roles in regulation of blood pressure^22,23^. Its role in pregnancy is unknown, but baseline serum RNLS levels are elevated during pregnancy^11^. Furthermore, consistent with other disorders including: systemic hypertension and renal disease, preeclampsia has been associated with reduced RNLS levels^6,10,11^. Recently, our group found that RNLS is endogenously produced by the placenta including in placental trophoblasts and Hoffbauer cells, resident placental macrophages^8^.

To date, no studies have assessed the role of RNLS in pathological placentas and while there are a few studies which have associated reduced serum RNLS and PEC^9–12^, it remains unknown whether this is different between preterm and term cases. Furthermore, it is unclear if either the native RNLS or acid sensitive RNLS fraction drives this reduction. As such we aimed to determine if there is an association between RNLS and PEC in placental tissue. Moreover, if the timing of the onset of PEC is related to either serum or placental levels of RNLS.

To determine if RNLS levels from maternal serum correlate with different presentations of PEC, we examined the acid sensitive and native RNLS fractions in preterm pregnancies with and without PEC and full-term pregnancies with and without PEC. An association of RNLS levels with PEC was only observed in preterm pregnancies where preterm serum samples affected by PEC had reduced levels of RNLS compared to preterm samples unaffected by PEC and were delivered for other indications. Interestingly, only the acid sensitive fraction was affected; the native form of serum RNLS was similar between cases with and without PEC. While the specific mechanisms resulting in PEC remain unknown, our finding that the acid sensitive fraction expressed in preterm preeclampsia samples was significantly lower than in preterm non-preeclampsia samples, in combination with the acid sensitive fraction accounting for the majority of maternally available RNLS in preterm samples, may imply that a deficiency in the acid sensitive RNLS fraction either leads to or can serve as a marker of more severe PEC in early pregnancy. Furthermore, given the observed association between PEC and lower serum RNLS levels, it is possible that the elevated blood pressure and heightened inflammatory states observed in PEC are due to decreased serum RNLS levels. This is potentially secondary to increased RNLS consumption as a compensatory mechanism of PEC. Alternatively, the reduced abundance of acid sensitive RNLS in the maternal serum of preterm preeclampsia samples may be partially explained by insufficient endogenous production of RNLS by various RNLS producing tissue.

Overall, the RNLS labeling was highest in the PV compared to both the decidua and the fetal membranes. When we examined the association between RNLS levels in various compartments of the placenta and PEC, we observed a reduction of RNLS associated with both preterm and term PEC within the decidua. However, the chorionic membrane expressed reduced levels of RNLS only in preterm cases, while the levels of RNLS within the placental villi were not affected by PEC. The observed reduction in RNLS levels in the maternal decidua and chorion in preterm cases of PEC suggested that the levels of RNLS are altered in PEC not only systemically but also locally in the placenta. Interestingly, levels of RNLS were reduced in the decidua in cases of PEC irrespective of gestational age, suggesting that local production or accumulation of RNLS at the maternal fetal interface is necessary throughout pregnancy and not just early gestation since even in term cases decreased levels of RNLS are associated with placental abnormalities. However, fetal membranes only demonstrated RNLS labeling reductions in preterm and not term cases, like the observations made in the maternal serum. This potentially suggests that RNLS activity is necessary throughout pregnancy at the site of implantation. Interestingly, we did not observe an association in RNLS levels in the PV neither in the trophoblasts or the interstitial with PEC in preterm or term cases even though the PV had the highest labeling for RNLS. This either suggests that PEC preferentially affects maternal rather than fetal tissue or that RNLS production within the PV is highly dynamic and is modulated in response to the increased consumption of RNLS in cases of PEC.

In summary, we show that RNLS in maternal serum and some components of placental tissue is associated with PEC. These changes may be indicative of PEC severity and would position RNLS as a potential marker for PEC diagnosis that should be evaluated in future studies.

## Methods

### Maternal serum procurement and processing

Maternal peripheral blood samples were collected by Yale University Reproductive Sciences (YURS) Biobank, centrifuged for serum which was removed and frozen prior to use. Informed consent was obtained from all subjects. The study was approved by the Yale Institutional Review Boards (IRB) (HIC#1309012696; IRB# 2000027134). All the experimental protocols were performed in accordance with relevant guidelines and regulations.

### ELISA

28 maternal serum samples from PP (n=7), PNP (n=6), TP (n=4), and TNP (n=11) patients were de-identified prior to distribution. Cohorts were balanced based on average gestation and sex (**Table S1**). ELISA was performed as we have described^21^. Plates were coated with m42-RNLS and incubated overnight at room temperature. Serum samples were either treated with or without 0.33M citric acid for 15 minutes followed by a neutralization step with 0.4M Na_2_PO_4_. Serum samples were added to the plates, diluted 1:1 with Diluent100, and incubated overnight at room temperature. Subsequent to 3 washes, a 1:1000 dilution of ab31291 was added to the plates and incubated at room temperature for 1 hour prior to 3 additional washes. Finally, a 1:1000 dilution of Sulfo-tagged anti-goat was used to indicate abundance RNLS antigen. RNLS values from samples without acid-treatment were defined as native RNLS whereas samples treated with acid were defined as acid sensitive RNLS. The latter may represent a circulating RNLS fraction that is tightly bound to other proteins or RNLS multimers.

### Placenta tissue procurement and processing

Placental samples were collected by Yale University Reproductive Sciences (YURS) Biobank, separated into the distinct layers of the placenta including: the decidua, chorion, and placental villi and formalin fixed. The biobank identified available cases of PP and TP and matched these to PNP and TNP based on sex and gestational age. The preeclampsia diagnosis was determined by the treating clinician. All samples without preeclampsia were subjects without any major hypertensive, metabolic disease, known congenital fetal anomalies, birth weight between 10^th^-90^th^ percentile, abnormal growth, placental abnormalities, infections, singleton gestation, nor illicit/licit substance use. Cohorts were balanced based on average gestational and sex (**Table S2**).

### Immunohistochemistry

19 formalin-fixed and paraffin-embedded placental samples from PP (n=5), PNP (n=4), TP (n=5), and TNP (n=5) patients were sectioned and mounted by Yale Histology Core. Slides were deparaffinized and rehydrated in ethanol prior to antigen retrieval using 10 mM citrate-buffered saline in a pressure cooker at 95°C for 20 minutes. Sections were washed with TBS-Tween (1%) with 300 mM NaCl prior to blocking with DAO Dual Endogenous Enzyme Block for Autostainer for 10 minutes followed by blocking with 2.5% horse serum for 1 hour (Agilent, Santa Clara, CA, USA). Slides were incubated with m28-RNLS (1:250 in TBS) overnight at 4°C and incubated in secondary antibody IMPRESS Reagent anti-Rabbit IgG for 45 minutes (Vector Laboratories, Burlingame, CA, USA). Antibody detection was performed with Vector’s ImmPACT DAB substrate kit prior to a hematoxylin counterstain (Vector Laboratories, Burlingame, CA, USA).

### Image Acquisition and Quantification

IHC images were acquired on the Echo Revolve microscope using 20x objectives. Post-acquisition, images were color-balanced in Adobe Photoshop to normalize the image backgrounds prior to color deconvolution in FIJI’s ImageJ using the H-DAB vector. The mean grey value (MGV) of each IHC image was quantified by exclusively selecting the relevant tissue section of each sample present in the high-powered field which served as a metric of RNLS abundance. 3 distinct images were captured for each sample and the MGV for each image was averaged to determine a representative RNLS value for each sample.

### Statistical Analysis

Statistical analysis was conducted using Prism GraphPad 8. Serum RNLS abundances (ng/mL) were compared using the one-way ANOVAs. The proportionality comparison between abundance of native RNLS to acid sensitive RNLS underwent a two-way ANOVA. All mean gray values (MGV) collected from the IHC images were compared using a non-parametric t-test (Mann-Whitney test) and significance was determined at a threshold of p ≤ 0.05. We accounted for sex-based differences within each cohort and observed a no significant sex driven differences. Additionally, average gestational age between preterm and term cohorts were consistent and demonstrated no statistically significant differences.

## Data Availability

The data that supports the findings present in this study are available upon request from the corresponding author.
